# The Effectiveness of the Back At work After Surgery (BAAS) Work-Integrated Care Pathway on Return to Work for Patients Receiving Knee Arthroplasty: A Study of Three Comparative Cohorts in the Netherlands

**DOI:** 10.1007/s10926-025-10331-1

**Published:** 2025-09-16

**Authors:** Daniël O. Strijbos, Geert van der Sluis, Wim F. C. van Houtert, A. Carlien Straat, Yvonne van Zaanen, Carolien M. Kooijman, Igor van den Brand, Stephan E. de Groot, Michiel F. Reneman, Tim Boymans, P. Paul F. M. Kuijer

**Affiliations:** 1 Department of Public and Occupational Health, Amsterdam UMC, University of Amsterdam, Meibergdreef 9, 1105 AZ Amsterdam, The Netherlands; 2 Department of Health Innovations, Nij Smellinghe Hospital Drachten, Compagnonsplein 1, 9202 NN Drachten, The Netherlands; 3 Hanze University of Applied Sciences Groningen, Zernikeplein 7, 9747 AS Groningen, The Netherlands; 4 Department of Orthopedics, Nij Smellinghe Hospital Drachten, Compagnonsplein 1, 9202 NN Drachten, The Netherlands; 5 Department of Orthopedics, Elizabeth Tweesteden Hospital, Doctor Deelenlaan 5, 5042 AD Tilburg, The Netherlands; 6 Elabo, Landdrostlaan 51, 7327 GM Apeldoorn, The Netherlands; 7 Department of Rehabilitation, University Medical Center Groningen, University of Groningen, Haren, The Netherlands; 8 Maastricht UMC +, Department of Orthopaedics, P. Debyelaan 25, 6229 HX Maastricht, The Netherlands

**Keywords:** Orthopedic procedures, Rehabilitation, Occupational medicine, Knee, Arthrosis

## Abstract

**Purpose:**

Considering the increase in the demand from working age patients seeking knee arthroplasty (KA) and the low return-to-work (RTW) rates, optimization of care for patients getting KA with a focus on RTW is essential. We evaluated a work-integrated care pathway—Back At work After Surgery (BAAS)—aimed at improving RTW compared with care-as-usual in the Netherlands.

**Methods:**

In this multicenter study of three comparative cohorts, working patients who had primary KA were included. Patients in two Dutch hospitals (BAAS cohort) received integrated medical and occupational care, including structured pre- and postoperative consultations, goal setting, activity tracking, and interdisciplinary team meetings with both medical and occupational health professionals. Two independent control cohorts with comparable patient inclusion criteria (Expect TO work and ACTIVE) from 15 hospitals/clinics received care-as-usual. The primary outcomes were the time to first day of RTW and time to full RTW within 12 months. Inverse Probability of Treatment Weighting was used with known prognostic factors as covariates to account for possible differences in baseline characteristics.

**Results:**

A total of 457 patients were included (BAAS *n* = 145; Expect TO work n = 179; ACTIVE *n* = 133). The median time to first day of RTW was 16–25 days shorter in the BAAS cohort (27 days) compared to Expect TO work (52 days; hazard ratio [HR] 2.7; 95% confidence interval [CI]:2.1–3.4) and ACTIVE cohort (43 days; HR:1.95; CI:1.5–2.6). At three months, 90% of BAAS patients had started RTW versus 63% and 77% in the control cohorts. BAAS patients also achieved full RTW earlier, with a median time reduced by 27 days compared to the ACTIVE cohort (HR:1.4; CI:1.1–1.8). The odds of full RTW at 12 months were higher in the BAAS cohort compared to Expect TO work, namely odds ratio (OR) 5.0 (CI:1.3–18.5) and ACTIVE OR 9.3 (CI:2.5–34.8).

**Conclusion:**

The BAAS work-integrated care pathway was more effective than care-as-usual in improving RTW after KA in the Netherlands.

**Trail Registration:**

This study was retrospectively registered at clinicaltrails.gov ( https://clinicaltrials.gov/ct2/show/NCT05690347, date of first registration: 19-01-2023).

**Supplementary Information:**

The online version contains supplementary material available at 10.1007/s10926-025-10331-1.

## Introduction

Work participation is a strong predictor of health, and should therefore be an important outcome measure in healthcare. Unfortunately work participation is often overlooked and taken for granted in practice and science [1–7]. In addition, working individuals contribute more effectively to society [8–11]. The importance of maintaining employment, therefore, contributes to valued individual and societal outcomes.

Care-as-usual often falls short in assisting individuals to stay at work or return to work (RTW) during or after an illness or medical condition [12–14]. This shortcoming requires that healthcare professionals should consider additional steps to secure work participation into their treatment [15]. Such additional steps for chronic low back pain have demonstrated that patients improved RTW with a median of 120 days (88 vs. 208) [16]. These additional steps can similarly be applied to common planned surgeries, like knee arthroplasty (KA). RTW after KA is a growing problem, due to the rising incidence of KA among patients in working age, and poor return to work outcomes [17–22]. Despite the favorable outcomes of KA in terms of pain and knee function, RTW among patients is low with reported worldwide non-RTW rates of 35% [13]. In the Netherlands, a similar non-RTW rate is seen with 31% [23]. Considering the increase in the demand from working age patients seeking KA and the low RTW rates after KA care, optimization of care for patients getting KA with a focus on RTW is essential.

To improve RTW after KA, a work-integrated care pathway named Back At work After Surgery (BAAS) was developed [24]. This pathway emphasizes a timely integration of medical and occupational care, enhancing patient participation throughout the perioperative clinical process with core components such as structured pre- and postoperative consultations, personalized goal setting, physical activity monitoring, and interdisciplinary meetings. BAAS’ feasibility in practice was demonstrated [25]. However, the effectiveness of BAAS for RTW was unknown. Our hypothesis was that the BAAS work-integrated care pathway is more effective on RTW than care-as-usual.

## Methods

### Study Design

We conducted a prospective cohort study evaluating the effectiveness of BAAS compared to two comparative cohorts studying care-as-usual as described in the Expect TO work cohort and ACTIVE trial [26, 27]. Ethical approval for the study was granted (reference IDs: W21_454#21.504 and L1429.2021). Reporting of this study adhered to both CONSORT and STROBE [28, 29] (See supplementary material, Appendix I).

### Participants and Study Setting

A minimal required sample size was calculated using R (version 3.6.3) with a power of 0.8 and a significance level of 0.05. The effect size was calculated with a mean difference of two weeks in comparison to a Dutch study on RTW after KA [30]. With a calculated minimal required sample size of 125 and an expected loss to follow-up of 20%, we intend to include 150 patients. Inclusion and exclusion criteria were similar for all three cohorts (See supplementary material, Appendix II). The recruitment procedure for BAAS is described in the protocol paper [24]. Patients were followed from recruitment before surgery until 12 months post-operatively or until full RTW was achieved. The BAAS study was performed in two hospitals in the Netherlands, Nij Smellinghe Hospital (NS) and Elizabeth Tweesteden Ziekenhuis (ETZ). These hospitals perform approximately 450 and 600 KAs annually [24]. The Expect TO work study was performed in seven Dutch hospitals, performing 430–770 KAs annually [27]. The ACTIVE trial was performed in eleven Dutch clinics and hospitals, performing 100–800 KAs annually [26].

### Patient and Public Involvement

To incorporate patient and public involvement, patients who had previously received KA, employers and an insurance company were consulted in the early development and feasibility testing of the BAAS work-integrated care pathway [25].

### Care-as-Usual

In the Netherlands, patients with symptomatic knee osteoarthritis (OA) are typically first managed with conservative treatments, such as physical therapy, pain medication, and lifestyle advice. Referral to an orthopedic surgeon and subsequent consideration for total knee arthroplasty (TKA) usually occurs when conservative treatment is no longer sufficient to relieve pain or restore function. According to national guidelines, an appropriate indication for TKA requires radiographic evidence of OA (e.g., Kellgren-Lawrence grade ≥ 2), significant limitations in daily life or work participation, and shared decision-making based on realistic expectations regarding outcomes and recovery [31]. Recovery after KA is clinically guided by a physical therapist who works in a hospital, while after discharge it is often guided by a primary care physical therapist who works accordingly to their professional guideline [32].

Parallel to the medical process, working patients in the Netherlands are legally supported by the Gatekeeper Improvement Act, which mandates early and structured RTW guidance after sick leave. Employers and occupational physicians are required to collaborate on a reintegration plan within six weeks of sick leave. The occupational physician evaluates work capacity and facilitates graded work resumption. In more complex cases, an occupational assessor may be involved to assess job demands, possibilities for workplace adaptations, or options for job transfers, often offered one year after sick leave. However, orthopedic care including rehabilitation and occupational support are typically not well integrated, and RTW is not a standard focus within usual perioperative orthopedic care.

### The BAAS Intervention

The BAAS work-integrated care pathway started with the indication of KA made together by the patient orthopedic surgeon, who actively refers the patient for a preoperative consultation by a hospital-based physical therapist. In this consultation, patients received counseling to optimize their preparation for surgery. Also, the patients completed several standardized questionnaires and performed functional tests to set a baseline measurement, which were used for evaluating clinical recovery [24]. A work-related therapy goal was set using Goal Attainment Scaling (GAS) [33]. Patients were asked to wear an accelerometer from 2 weeks preoperatively (PAM 2.0, PAM B.V., Oosterbeek, the Netherlands) to monitor physical activity with real-time feedback accessible to patients and healthcare professionals via the Atris app (Peercode, Geldermalsen, the Netherlands). An occupational assessor compiled a report of beneficial and limiting factors regarding RTW based on a preoperative workplace assessment (See supplementary material, Appendix III).

Around the fourth or fifth week after surgery, an online team meeting was held involving the patient, primary care physical therapist, hospital-based physical therapist, occupational assessor, and occupational physician. This interdisciplinary consultation focused on evaluating the recovery progress and using this information to refine the RTW plan, if necessary. Next to this online interdisciplinary consultation, postoperative consultations by the hospital were organized at 6 weeks and every 3 months thereafter, up to 12 months or until full RTW was achieved. During these consultations, recovery was assessed by monitoring the GAS goal, and by using the same questionnaires and functional tests as those at baseline.

Postoperative care included targeted physical therapy in a primary care setting, adhering to the Royal Dutch Society for Physiotherapy guidelines for knee osteoarthritis [32]. If the hospital-based physical therapist expected that a patient could not RTW within 12 months —based on patient experience, clinical assessments, and accelerometer data—patients were referred for a multidisciplinary rehabilitation assessment in an academic hospital. This assessment could lead to participation in a vocational rehabilitation program.

### Outcome Measures

#### Primary Outcomes

The primary outcomes were the first day of RTW and the first day of full RTW [34]. The first day of RTW was defined as the number of calendar days from surgery until the patient returned to work, regardless of the number of hours worked or type of tasks performed. Full RTW was defined as the number of calendar days from surgery until the patient resumed working the same number of hours as specified in their employment contract, regardless of the tasks performed. To the best of our knowledge, there is no previous study concerning minimal clinical differences (MCID) in return to work after surgery. Therefore, we held a consensus meeting with authors DS, GS, TB, PK, and MR in which we concluded that a minimal clinical difference of two weeks on start to return to work and one week for full return to work is relevant [24]. RTW outcomes were self-reported in all three cohorts. Because the Expect TO work cohort measured RTW as a binary outcome (yes/no) at 3, 6, and 12 months, these data were incorporated to further refine and enhance the analysis compared to what was originally outlined in the protocol.

#### Secondary Outcomes

The secondary outcomes were the KOOS and WORQ at baseline (preoperative), and at follow-up until full RTW was achieved [35, 36]. The KOOS is a 42-item, 5-point Likert scale questionnaire which can be calculated in five 0–100 domain scores [36]. The domains are: Pain, Other Symptoms, Activities of daily Living (ADL), Sports/Recreation and Quality of Life (QoL). The MCID of each domain is 12, 9, 10, 9, and 14 points, respectively [32]. The WORQ is a 13-item, 5-point Likert scale questionnaire that assesses the perceived difficulty of work-related activities and is calculated on a 0–100 scale. The WORQ has a MCID of 13 points [35].

#### Demographics

Data on age, sex, BMI, type of surgery, and comorbidities were obtained from the electronic patient records. Working hours per week, physical demands of the job, preoperative sick leave, being breadwinner, level of education, and being self-employed were asked to the patient in the preoperative consultation.

### Statistical Methods

The statistical analysis tested the effectiveness of the BAAS work-integrated care pathway on RTW outcomes, compared with the comparative care-as-usual cohorts. The statistical methods deviated from the protocol paper, due to the fact that a uni- or multivariate linear regression was, in hindsight, not the most robust statistical method to analyze a time-to-event outcome. All analyses were conducted using R (version 4.1.0).

#### Inverse Probability of Treatment Weighting (IPTW) to Estimate Time to RTW

To account for possible differences in baseline characteristics of the patients between the intervention and control cohorts, propensity scores were calculated using logistic regression. The relevant covariates for RTW were sex (female vs. male), type of surgery (UKA vs. TKA), BMI, physical demands of the job (knee-straining vs. non-knee-straining job), preoperative sick leave (yes vs. no), and the self-reported work-relatedness of the knee arthroplasty (ranging from ‘totally agree’ to ‘totally disagree’) [33, 34]. If missing data were present in the covariates, Predictive Mean Matching via the mice package was used with five imputations to ensure robustness of the estimates. These propensity scores were used to derive inverse probability of treatment weights (IPTW), which adjusted for covariates in the regression models without performing direct matching. This method ensured that the weighted samples more closely resembled a randomized comparison between BAAS and the control cohorts. The effectiveness of BAAS on first day and full RTW was assessed using hazard ratios (HRs) including 95% confidence intervals (95%CIs) using Cox regression models. Difference in time to first day and full RTW was assessed by calculating the median including interquartile ranges (IQRs).

#### Logistic Regression for Proportions

Logistic regression models were used to assess the proportion of patients who succeeded in first day of RTW and full RTW at 3, 6, 9, and 12 months, with odds ratios (ORs) and IQRs calculated for the intervention versus control groups. Firth’s bias-reduced logistic regression was used to mitigate bias in small sample sizes.

#### Secondary Outcomes

For the secondary outcomes, mean changes in the five KOOS domains and the WORQ score were calculated for the three cohorts. A repeated measures ANOVA was performed with time (preoperative and three months postoperative) as the within-subject factor and cohort as the between-subject factor. If there was a main effect of cohort, post hoc Bonferroni analysis assessed the effect of each cohort individually in comparison to BAAS.

#### Sensitivity Analyses

A sensitivity analysis was performed to determine whether outcomes differed between the two study hospitals (NS and ETZ). For each center, multivariate Cox proportional hazards models were fitted to estimate the effect of BAAS on both time to first day of RTW and time to full RTW. Median survival times for first day and full RTW were calculated for both the NS and ETZ hospitals, and interquartile ranges (IQR) were determined to assess the variability in median differences across centers.

## Results

A total of 457 patients were included from three cohorts: BAAS (*n* = 145), Expect TO work (*n* = 179), and ACTIVE (*n* = 133) (See supplementary material, appendix IV). Missing data on the covariates—work-relatedness of knee OA (*n* = 37), sick leave (*n* = 36), BMI (*n* = 6), and physical job demands (*n* = 1)—were imputed for a total of 38 patients (8%). Two patients were excluded from the NS hospital cohort due to postponed surgery or due to termination of the work contract, respectively. From the 145 remaining patients, nine dropped out: eight patients were unwilling and one patient had additional surgery within the RTW period. Within the first year after surgery, 139 patients started RTW and 136 fully RTW. The characteristics of the cohorts did not differ significantly for sex, BMI, and preoperative sick leave. Significant differences were observed for type of surgery, working in a knee-straining job and level of education (Table 1).

**Table 1:** Patient baseline and perioperative characteristics. Data are numbers (n) and percentages (%) of patients unless stated otherwise

	BAAS (*n* = 145)	Expect TO work (*n* = 179)	ACTIVE Trial (*n* = 133)	*p*
Mean (SD) age (years)	58.4 (4.3)	57.9 (5.2)	58.7 (4.4)	0.308
Female sex	77 (53.1)	92 (51.4)	69 (51.9)	0.953
Mean (SD) BMI	29.4 (3.9)	29.8 (5.1)	29.7 (4.0)	0.701
Type of surgery (TKA)	115 (79.3)	179 (100)	88 (66.2)	< 0.001
Comorbidities				0.150
0	115 (82.7)	149 (83.2)	103 (77.4)	
1	20 (14.4)	25 (14.0)	20 (15.0)	
> 1	4 (2.9)	5 (2.8)	10 (7.5)	
Knee-straining job (yes)	72 (50.0)	89 (49.7)	38 (28.6)	< 0.001
Education				< 0.001
No	4 (2.9)	4 (2.7)	0 (0.0)	
Primary	29 (20.7)	15 (10.1)	11 (8.3)	
Lower secondary	76 (54.3)	69 (46.3)	59 (44.4)	
Higher secondary	12 (8.6)	14 (9.4)	9 (6.8)	
Higher vocational	18 (12.9)	36 (24.2)	48 (36.1)	
University or higher	1 (0.7)	11 (7.4)	6 (4.5)	
Breadwinner (yes)	80 (57.6)	113 (63.1)	81 (60.9)	0.600
Self-employed (yes)	27 (18.9)	24 (13.5)	25 (18.8)	0.328
Sick leave (yes)	30 (21.4)	36 (24.3)	27 (20.3)	0.700
Mean (SD) contract hours	31.3 (12.7)	31.8 (9.7)	32.4 (19.1)	0.833

*BAAS* Back At work After Surgery, *BMI* Body Mass Index, *SD* standard deviation, *TKA* total knee arthroplasty

### Time to First day of RTW and Full RTW

**Fig. 1:**
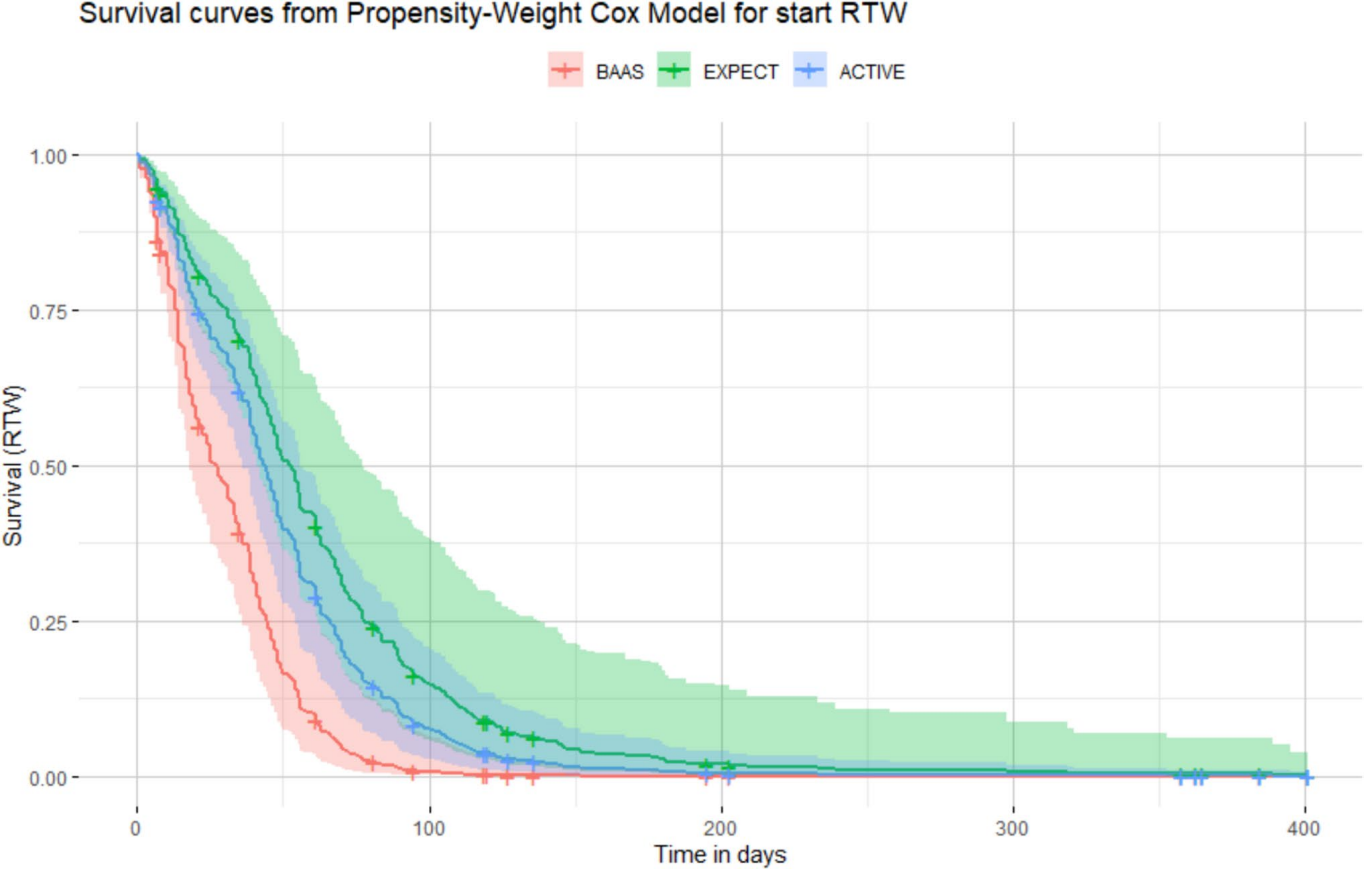
Survival curve of IPTW-adjusted Cox model for first day of RTW with 95% confidence interval for the cohorts BAAS, Expect TO work and ACTIVE. *BAAS* Back At work After Surgery

**Fig. 2:**
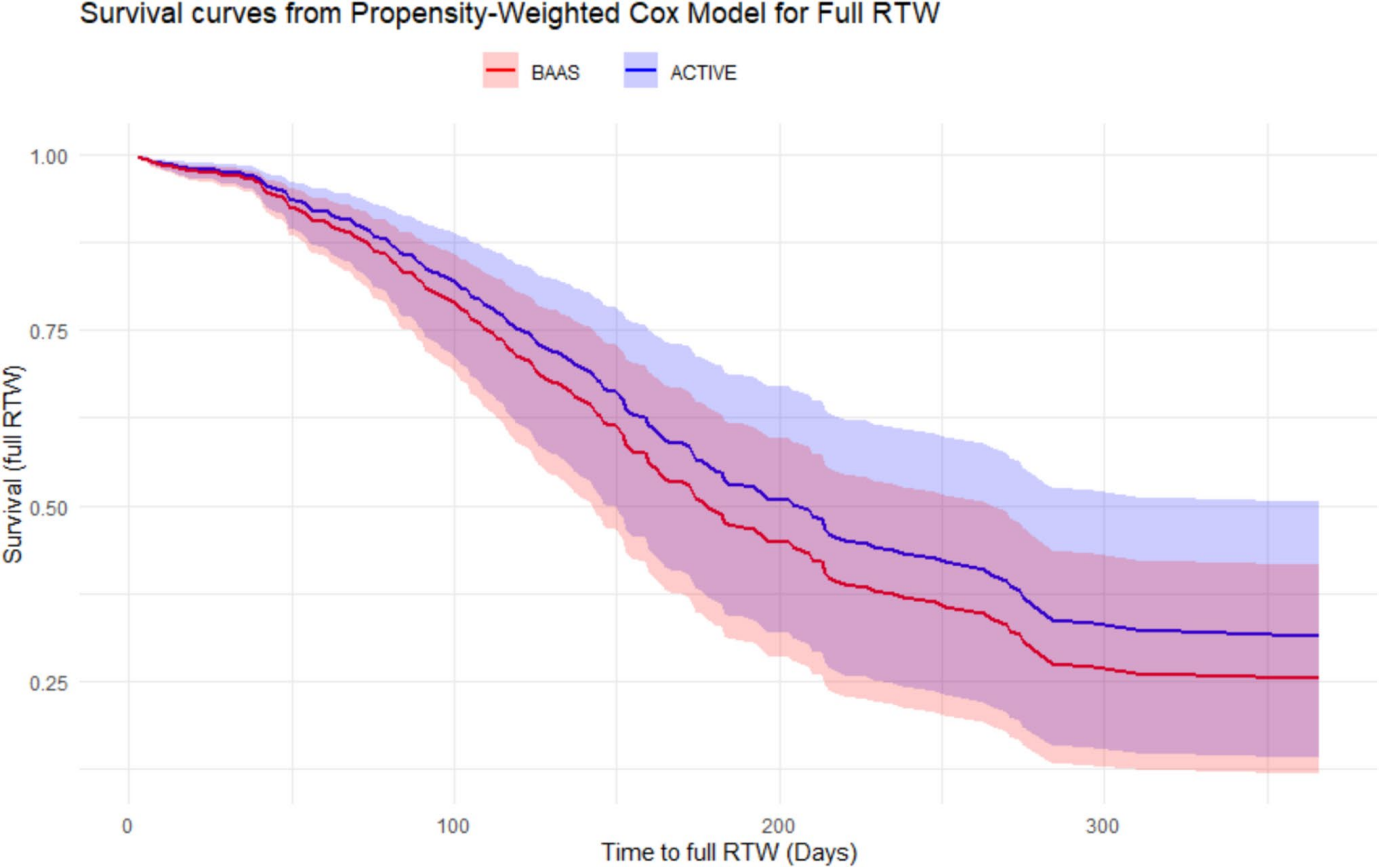
Survival curve of IPTW-adjusted Cox model for full RTW with 95% confidence interval for the cohorts BAAS and ACTIVE. Abbreviations: *BAAS* Back At work After Surgery

The BAAS cohort had an earlier first day of RTW compared to the care-as-usual cohorts Expect TO work (HR = 2.7, 95%CI 2.1–3.4, *p* < 0.001) and ACTIVE (HR = 1.95, 95%CI 1.5–2.6) with a median of 27 days (IQR: 14–44) versus 52 (IQR: 21–66) and 43 (IQR: 31–78), respectively (Fig. 1). Therefore, the absolute median difference between BAAS and Expect TO work is 25 days, and between BAAS and ACTIVE 16 days. For full RTW, the median was 183 days (IQR: 115– > 365 (within this analysis, > 25% did not fully RTW, see Fig. 1)) for BAAS and 210 days (IQR: 124– > 365) for ACTIVE, giving an absolute median difference of 27 days (HR = 1.4, 95%CI 1.1–1.8) (Fig. 2).

### Proportions Over Time for First and Full RTW

In the BAAS cohort, more patients (93%) started with RTW within three months, compared to Expect TO work (64%) and ACTIVE (77%). Logistic regression models with Firth’s bias-reduction method indicated significant differences in start to RTW rates across cohorts over time in favor of BAAS, with ORs ranging from 5.3 to 14.8, respectively (Table 2). BAAS showed that more patients started to RTW compared to Expect TO work at 3, 6, 9, and 12 months after KA, and also at 3 and 6 months after KA compared to ACTIVE. Differences between BAAS and ACTIVE at the 9 and 12 months were non-significant.

**Table 2:** Odds ratios for proportions of patients starting RTW and full RTW between the cohorts BAAS, Expect TO work and ACTIVE

Time point	Start RTW		Full RTW	
	BAAS vs Expect TO work (1/OR [95% CI])	BAAS vs ACTIVE (1/OR [95% CI])	BAAS vs Expect TO work (1/OR [95% CI])	BAAS vs ACTIVE (1/OR [95% CI])
**3 months**	8.0 [3.9–18.2]	7.3 [3.2–17.7]	1.4 [0.8–2.4]	0.9 [0.5–1.9]
**6 months**	6.8 [1.6–64.0]	7.4 [1.5–73.1]	2.9 [1.6–5.2]	2.5 [1.3–4.8]
**9 months**	14.8 [1.7–1951]	9.4 [0.9–1276]	*Data not available*	4.4 [1.7–11.6]
**12 months**	11.0 [1.3–1423]	5.3 [0.3–794]	5.0 [1.3–18.5]	9.3 [2.5–34.8]

*BAAS* Back At work After Surgery

For full RTW, in the BAAS cohort, 4 (2%) patients did not fully RTW within 12 months, compared to 22 (16%) and 17 (13%) in the Expect TO work and ACTIVE cohort, respectively. Differences in full RTW odds at three months were non-significant. However, the BAAS cohort demonstrated significantly higher odds of patients who successfully fully RTW from six to twelve months post-surgery, with ORs ranging from 2.5 to 9.3.

### Sensitivity Analysis

The median time to start RTW was 43 days (IQR: 19–62) for NS hospital and 42 days (IQR: 18–60) for ETZ hospital, with a median difference of 1 day. For full RTW these data were 110 days (IQR: 75–153) for NS hospital, and 136 days for ETZ hospital (IQR: 89–192), with a median difference of 26 days.

### Secondary Outcomes

**Fig. 3:**
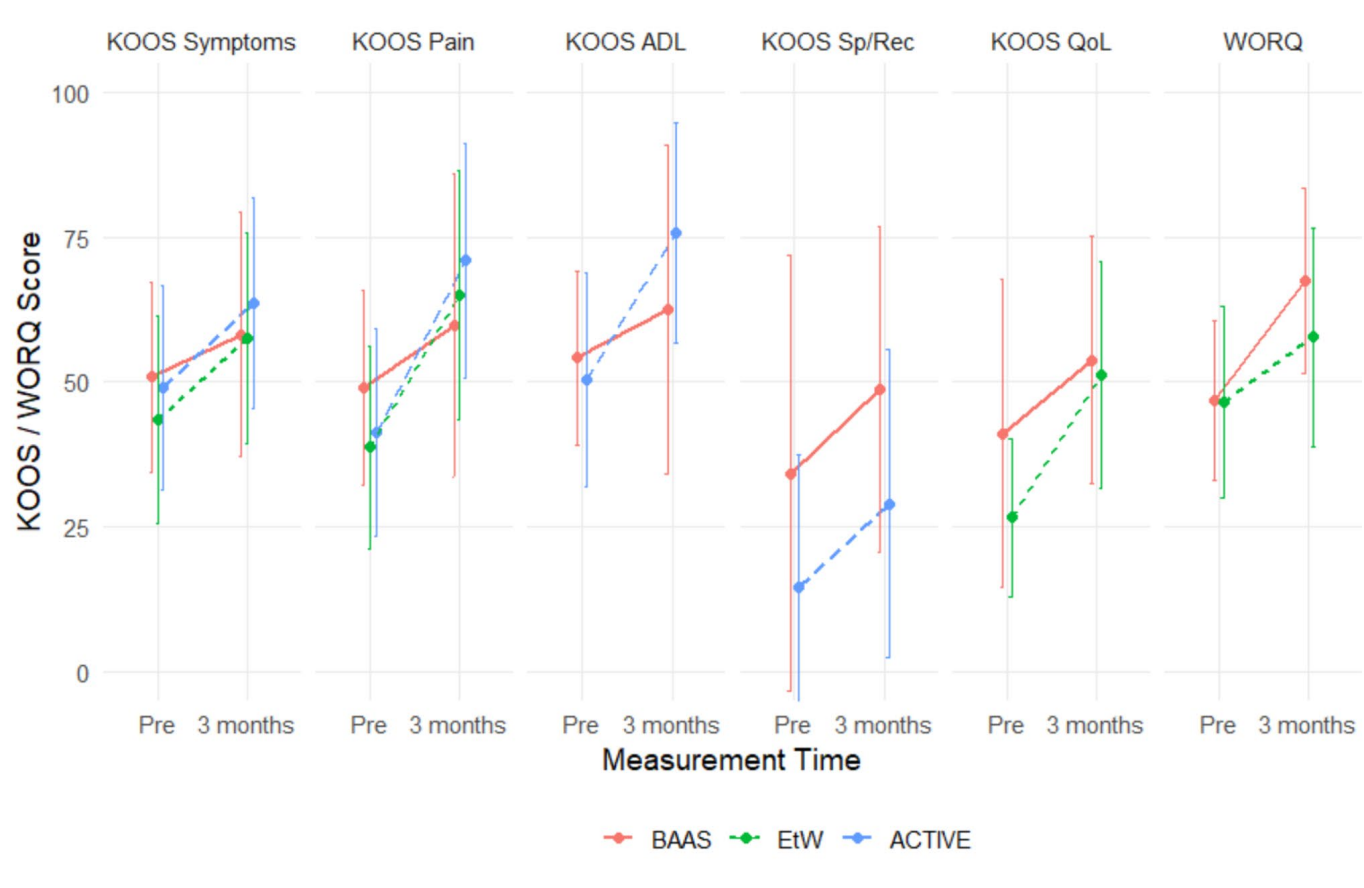
Mean scores (SD) for the Knee Osteoarthritis Outcome Score and the Work, Osteoarthritis and joint-Replacement Questionnaire in time (preoperative and 3 months after surgery) for the cohorts BAAS, Expect TO work and ACTIVE. *ADL* activities of daily living, BAAS Back At work After Surgery, *EtW* Expect TO Work, *KOOS* knee osteoarthritis outcome score, *QoL* quality of life, *Sp/Rec* sports and recreation, *WORQ* Work, Osteoarthritis and joint-Replacement Questionnaire

Given that most patients in the BAAS cohort achieved full RTW before 6 months, only the preoperative and 3 months data are presented. All KOOS domains and the WORQ outcomes showed significant improvement over this time period across all three cohorts (*p* < 0.001) (Fig. 3 and See supplementary material, appendix V). Recovery for the WORQ and the following KOOS domains favored BAAS: Sports & Recreation and Quality of Life. Recovery in the following KOOS domains favored care-as-usual, exceeding the MCID, whereas BAAS did not: KOOS symptoms (only in comparison to Expect TO work) and ADL (See supplementary material, appendix V).

## Discussion

In this multicenter study of three comparative cohorts, we found that the BAAS work-integrated care pathway is more effective for RTW compared to two care-as-usual cohorts. Secondary outcomes showed clinical advantages favoring care-as-usual and participation benefits favoring BAAS, illustrating that meaningful participation (including RTW) can be achieved even when clinical outcomes are not fully optimal [37].

Our study has several strengths. First, it included a large number of patients, increasing reliability. Second, the intervention was implemented in everyday clinical practice in two hospitals, thereby increasing its generalizability. Third, the work-integrated care pathway was personalized, as each patient’s recovery was monitored, goals were set using Goal Attainment Scaling, and individual work activities were discussed and taken into account. Finally, the observed time-to-RTW benefits (16–25 days earlier for start RTW and 27 days for full RTW) exceeded MCIDs [24].

To assess generalizability, comparison to another Dutch care-as-usual suggests the time to start RTW can be longer (84 days, SD 54) than in our two care-as-usual cohort namely 52 and 43 days [30]. A recent meta-analysis based on 25 studies from nine countries reported a pooled mean time to RTW of 90 days (range 35–205) [13]. BAAS with 27 days seems to outperform these care-as-usual studies. However, a reliable comparison is hard to make due to two reasons. First, there is no clear definition whether first or full RTW was measured. Second, RTW might have been calculated only for those who did RTW within the study period, instead of measuring RTW for all included patients as is done in the present study. Regarding the proportion of patients with no RTW within one year, BAAS seems to outperform the international comparison with 2% versus 35% [13]. However, the inclusion criteria are probably not the same for instance regarding the intention to RTW after surgery.

We are aware of one other study evaluating a work-integrated care intervention after KA, namely the Finnish Coordinated Return to Work (CRTW) study [38, 39]. Patients receiving CRTW started RTW after a mean of 87 days (SD 52) compared to a median of 27 days in our BAAS-intervention cohort [39]. Also, 13% of the patients did not RTW in the CRTW study within one year, compared to 2% in the BAAS cohort [39]. The CRTW study, like BAAS, reported a positive result for the intervention group with a mean difference of 14.4 days (95% CI 21.3 to 7.5) compared to a median difference of 16–25 days in our study [38]. Although these outcomes cannot be perfectly compared (mean vs. median), the difference is notable. Like the systematic review with meta-analysis, the CRTW study seemed to only report time to RTW for those who did RTW and not for the patients with no RTW [13, 38, 39].

Our study contributes to the growing evidence supporting work-integrated healthcare. Thereby, not only patients benefit but also society by reducing work absenteeism. This is particularly important in KA given the current rise among patients of working age [17–22]. BAAS prioritizes participation over solely improving physical function or reducing pain. This approach aligns with patients’ experiences of recovery, which are often based on meaningful activities such as work, sports, and leisure, and poses a challenge for healthcare professionals who traditionally focus on bodily functions and structures and clinical signs [40, 41]. Furthermore, the results underscore the critical need to explore whether BAAS can similarly benefit other orthopedic procedures, like hip arthroplasty and discectomy, highlighting the profound societal value of work-integrated health care [13, 42]. Lastly, an economic evaluation showed that, in addition to being effective as demonstrated in this study, BAAS also yielded a positive return on investment of over 500% from both the societal and worker’s perspective [43].

Nevertheless, four potential limitations merit consideration. First, our study design was not a randomized controlled trial (RCT). However, the two care-as-usual cohorts were comparable regarding eligibility criteria, study period, clinical setting, RTW outcomes and key covariates. Moreover, the application of IPTW adjusted for confounders for RTW, strengthening the validity of our findings. In addition, a comparison with comparable cohorts reduced both time and costs compared to an RCT, and potentially enhanced the validity of the results [44, 45]. Second, sensitivity analyses revealed that the hospital where the lead scientist directly provided part of the care (NS) achieved better full RTW outcomes compared to the other hospital (ETZ). This may be due to more extensive focus and experience of the care team. The implementation in ETZ hospital consisted of only a one-day training and a phone call every two weeks, which might have been too little. Third, RTW was measured by self-reports, which could introduce reporting bias. However, recent evidence shows that the discrepancy between registry studies and self-reported data regarding RTW are minimal, with a pooled mean difference of only 1.8 sickness absence days and for proportions a sensitivity of 0.83 and a specificity of 0.92 [46]. Also, our outcomes are in line with the recently published Core Outcome Set for Work participation (COS for Work) [34]. Lastly, Third, the BAAS intervention cohort contained proportionally more participants with a lower level of education, even after matching (Table 2). Lower level of education has been linked to slower functional recovery and a longer time to RTW after KA [47–50]. Because education is highly correlated with physical job demands, we included only the “knee-straining job” variable in the propensity model to avoid multicollinearity, and this characteristic was balanced between groups. Residual confounding by education can therefore not be ruled out; if lower-educated workers indeed face a poorer prognosis, however, the current cost advantage observed for BAAS is likely an underestimate compared to a better education-balanced situation.

In conclusion, this multicenter study of three comparative cohorts demonstrates that the BAAS work-integrated care pathway is more effective than care-as-usual in improving return to work after KA in the Netherlands. Patients returned to work sooner and more frequently, despite comparable clinical outcomes. These findings underscore the relevance of integrating occupational support within perioperative care, especially for patients of working age. BAAS emphasizes meaningful participation, aligned with patients’ recovery goals, without compromising medical recovery. Further research is needed to evaluate its applicability in other surgical populations and healthcare contexts.

## Supplementary Information

Below is the link to the electronic supplementary material.

Supplementary file1 (DOCX 755 kb)

## Data Availability

The datasets generated during and/or analyzed during the current study are available from the corresponding author on reasonable request.
